# Five novel mutations in steroidogenic factor 1 (SF1, *NR5A1*) in 46,XY patients with severe underandrogenization but without adrenal insufficiency

**DOI:** 10.1002/humu.20588

**Published:** 2008-01

**Authors:** Birgit Köhler, Lin Lin, Bruno Ferraz-de-Souza, Peter Wieacker, Peter Heidemann, Vanessa Schröder, Heike Biebermann, Dirk Schnabel, Annette Grüters, John C Achermann

**Affiliations:** 1 Department of Pediatric Endocrinology, University Children's Hospital, Charité, Humboldt UniversityBerlin, Germany; 2 University College London Institute of Child Health, University College LondonLondon, United Kingdom; 3 Institute of Human Genetics, Otto-von Guericke Universitiy of MagdeburgMagdeburg, Germany; 4 Children's Hospital AugsburgAugsburg, Germany

**Keywords:** steroidogenic factor-1, SF1, *NR5A1*, gonadal dysgenesis, disorders of sex development (DSD), male pseudohermaphroditism, nuclear receptor

## Abstract

Steroidogenic factor 1 (SF1, *NR5A1*) is a nuclear receptor that regulates multiple genes involved in adrenal and gonadal development, steroidogenesis, and the reproductive axis. Human mutations in SF1 were initially found in two 46,XY female patients with severe gonadal dysgenesis and primary adrenal failure. However, more recent case reports have suggested that heterozygous mutations in SF1 may also be found in patients with 46,XY partial gonadal dysgenesis and underandrogenization but normal adrenal function. We have analyzed the gene encoding SF1 (*NR5A1*) in a cohort of 27 patients with 46,XY disorders of sex development (DSD) from the German network of DSD. Heterozygous SF1 mutations were found in 5 out of 27 (18.5%) of cases. Four patients with SF1 mutations presented with the similar phenotype of mild gonadal dysgenesis, severe underandrogenization, and absent Müllerian structures. Of these, two patients harbored missense mutations within the DNA-binding region of SF1 (p.C33S, p.R84H), one patient had a nonsense mutation (p.Y138X) and one patient had a frameshift mutation (c.1277dupT) predicted to disrupt RNA stability or protein function. One additional patient ([c.424_427dupCCCA]+[p.G146A]) displayed a more marked phenotype of severe gonadal dysgenesis, normal female external genitalia, and Müllerian structures. Functional studies of the missense mutants (p.C33S, p.R84H) and of one nonsense mutant (p.Y138X) revealed impaired transcriptional activation of SF1-responsive target genes. To date, adrenal insufficiency has not occurred in any of the patients. Thus, SF1 mutations are a relatively frequent cause of 46,XY DSD in humans.

## INTRODUCTION

Steroidogenic factor–1 (SF1/Ad4BP, *NR5A1*; MIM# 184757) is a nuclear receptor that was first identified following the search for a common regulator of the cytochrome P450 steroid hydroxylase family of enzymes [Lala et al., 1992]. Subsequent studies have revealed that SF1 regulates a great number of genes involved in gonadal and adrenal development, reproduction and steroidogenesis [Parker et al., 2002].

SF1 is believed to bind to the response elements of target genes as a monomer. Critical regions of SF1 involved in transcriptional regulation include a two zinc finger DNA–binding domain (DBD), an “A” box region found in monomeric nuclear receptors, a hinge region, and a helical ligand–binding domain (LBD) region containing an activation function–2 (AF–2) domain [Parker and Schimmer, 1997]. Crystalization of the LBD of SF1 has revealed that phospholipids may act as ligands for SF1 [Krylova et al., 2005].

SF1 is essential for gonadal and adrenal differentiation in mammals. In mice and humans, SF1 is expressed very early in the urogenital ridge and continues to be highly expressed in the developing adrenal, gonad, ventromedial hypothalamus, and pituitary [Hatano et al., 1994; Ingraham et al., 1994; Hanley et al., 1999]. Targeted disruption of Sf1 (*Ftzf1*) in mice results in gonadal and adrenal agenesis, persistence of Müllerian structures and abnormalities of the hypothalamus and pituitary gonadotropes [Luo et al., 1994; Majdic et al., 2002]. Heterozygous animals have a milder phenotype including an impaired adrenal stress response and reduced testicular size [Bland et al., 2000].

In humans, SF1 mutations were first described in patients with 46,XY disorders of sex development (DSD), Müllerian structures, and primary adrenal failure (MIM# 184757) [Achermann et al., 1999, 2002]. The first of these mutations (p.G35E) was a de novo heterozygous change affecting a critical amino acid in the “P” box region of the first zinc–finger of the DNA binding domain that severely affects SF1 function [Achermann et al., 1999; Ito et al., 2000]. The second mutation was a recessively–inherited homozygous change (p.R92Q) affecting the “A” box of SF1 and causing partial loss of function [Achermann et al., 2002]. A heterozygous SF1 mutation has also been reported in a 46,XX female with primary adrenal failure and apparently normal ovarian development [Biason-Lauber and Schoenle, 2000].

Recently, heterozygous SF1 mutations have been described in seven 46,XY patients with ambiguous genitalia, gonadal dysgenesis, but no adrenal insufficiency [Correa et al., 2004; Mallet et al., 2004; Hasegawa et al., 2004; Lin et al., 2007]. The potential dosage–dependent actions of SF1 suggest that these heterozygous SF1 changes might lead to a partial phenotype of 46,XY DSD with normal adrenal function. Here, we report five novel SF1 mutations identified in a cohort of 27 patients with severe underandrogenization and variable degrees of gonadal dysgenesis. These results suggest that heterozygous SF1 changes are a relatively frequent finding in patients with 46,XY gonadal dysgenesis/DSD.

## MATERIALS AND METHODS

### Patients

The cohort studied consisted of 27 46,XY patients from the “German Network of Disorders of Sex Development.” The patients had presented with phenotypes ranging from ambiguous genitalia to normal female genitalia and had been diagnosed with variable degrees of gonadal dysgenesis. Müllerian structures were reported in 17 patients. Adrenal insufficiency was not present in any of them. Dysmorphic or systemic features (e.g., kidney, heart, limbs) were absent. Informed consent for the genetic studies was obtained from all parents or patients according to institutional guidelines.

### Molecular Analysis of NR5A1

Genomic DNA was extracted from peripheral blood leukocytes and exons 2–7 of the gene encoding SF1 (*NR5A1*) were PCR–amplified (primer sequences available on request). PCR products were purified using exonuclease 1 and shrimp alkaline phosphatase (New England Biolabs, Cambridge, UK; USB, Cleveland, OH) and sequenced using a V3 kit and 3130_x_ analyzer (Applied Biosystems, Foster City, CA). DNA mutation numbering is based on GenBank reference DNA sequence NM_004959.3, with the A of the ATG initiation codon designated +1 (www.hgvs.org/mutnomen).

### Eletromobility Shift Assays

Wild–type (WT) and mutant SF1 proteins were in-vitro translated from the _p_CMX expression vector using the TNT reticulocyte system (Promega, Southampton, UK). Synthetic oligonucleotide probes corresponding to the 30 SF1 binding site on the *Cyp11a* minimal promoter were labeled with [^32^P]dCTP by Klenow polymerase and electromobility shift assays were performed as described previously [Ito et al., 2000].

### Studies of SF1 Expression and Nuclear Localization

Mutant green fluorescent protein (GFP)–SF1 constructs were generated by site–directed mutagenesis in a pAcGFP–C1 vector (Clontech, Oxford, UK) to produce mutant fusion proteins with a GFP tag at the amino–terminus of SF1. Studies of protein expression and cellular localization were performed by transfecting WTand mutant pAcGFP–C1SF1 expression vectors (0.8 µg) into tsa 201 human embryonic kidney cells. After 24 hr, cells were fixed and nuclear counterstaining performed with Vectashield containing DAPI (Vector Laboratories, Peterborough, UK).

### Transient Gene Express Assays

Expression vectors containing the _p_.C33S and _p_.R84H missense mutants and _p_Y138X nonsense mutant were generated by sitedirected mutagenesis (QuikChange, Stratagene, Amsterdam, The Netherlands) using WT _p_CMXSF1 as a template. Transient transfection studies were performed in 96–well plates using Lipofectamine 2000 (Invitrogen, Paisley, UK) and a Dual–Luciferase reporter assay system (Promega). Initial studies were performed in tsa 201 cells by transfecting empty, WT, or mutant SF1 expression vectors (2ng/well) (p.G35E, p.C33S, p.R84H, p.Y138X) with SF1–responsive minimal promoters linked to luciferase (100ng/well) (data shown for *Cyp11a*). In addition, synergistic activation of the LHβ promoter by SF1 and Egr1 (2ng/well) was studied in tsa 201 cells, as described previously [Lin et al., 2007]. Finally, WT or mutant SF1 vectors (10ng/well) were cotransfected with *Cyp11a* and MIS reporters into TM3 (mouse Leydig) and TM4 (mouse Sertoli) cells, respectively. Cells were lysed for 24 hr following transfection and assayed for luciferase activity (FLUOstar Optima; BMG Labtech, Aylesbury, UK), with standardization for *Renilla* coexpression.

### Studies of Potential Dominant Negative Interactions

Studies of WT/mutant interactions were performed by transfecting increasing amounts of _p_CMXWT or mutant SF1 expression vector (0, 1, 2, 5, 10 ng) with either 1 ng empty vector or 1 ng WT SF1 and *Cyp11a* reporter (100 ng) in tsa 201 cells. Luciferase assays were performed as described above.

## RESULTS

### Cohort Analysis

**FIGURE 1:**
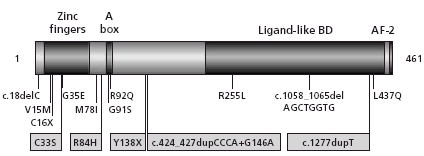
Structure of SF1 including the position of SF1 mutations in 46,XYpatients with disorders of sex development. p.G35E and p.R92Q are the first mutations described in SF1 in 46,XY individuals with gonadal dysgenesis and adrenal failure. p.R255L was found in a normal 46,XX female with adrenal failure.The othermutations have been identified in individuals with 46,XY DSD without adrenal insufficiency.The five novel mutations reported here are boxed. DNA RefSeq NM_004959.3.

SF1 mutations were found in 5 out of 27 (18.5%) patients studied. An overview of these changes and clinical phenotypes is provided in Table 1 and Figure 1.

**TABLE 1:** Phenotypes, Genotypes, and Hormonal Data of the FIve Patients*

Patient	Age (years)	External genitalia	Uterus	Epididymis and vas deferens	Gonada histology	Testosterone levels at diagnosis	Gender assignment	Adrenal investigations (at current age)	Mutation	Parents
1	4	Clitoromegaly urogenital sinus, inguinal gonads	No	Yes	Bilateral testes, Sertoli rich, few spermatogonia and Leydig cells	hCG stimulation at birth: testosterone <0.08→0.07 ng/ml	♀	Cortisol 3.6 µg/dl DHEAS 50 ng/ml ACTH 25 pg/ml	_p_.C33S(_c._ 98G>C, exon 2)	Mother: normal; father: normal
2	17	Citoromegaly urogenital sinus, inguinal gonads	No	Yes	Bilateral testes	hCG stimulation at 2 years: testosterone <0.01→2.5ng/ml	♀	Corsitol 7.4 to 24.9µg/dl^a^DHEAS 1230 ng/ml	_p_.R84H (_c_.251G>A, exon 4)	Mother: normal; father; NA
3	10	Clitoromegaly, urogenital sinus inguinal gonads	No	Yes	Bilateral testes Sertoli cells only	At birth: testosterone 0.25ng/ml	♀	Cortisol 7.2µg/dl DHEAS 891ng/ml	_p_Y138X(_c_414C>A, exon 4)	Mother: normal; father; NA
4	8	Clitoromegaly urogenital sinus, inguinal gonads	No	Yes	Bilateral testes	hCG stimulation at birth;testostrrone 0.86→.086ng/ml	♀	Cortisol 6.7 µg/dl DHEAS 548ng/ml	_c_1277dupT(exon 7)	Mother: ^c^1277 dupT; father: NA
5	22	Normal female, no aplpable gonads	Yes	Not known	Bilateral streak gonads	At 22 years: LH 25.8U/I FSH 111.9U/I estradiol 7pg/ml testosterone 0.2 nd/ml	♀	NA	_c_424_427 dupCCCA+_p._G146A polymorphism(_c._437G>C)(both exon 4)	Mother: NA; father; NA

* Normal ranges: basal cortisol, 3–15 µg/dl (<0.5–10 years); DHEAS, <50–352 ng/ml (0.5–7 years), 75–1,312 ng/ml (7–10 years), 150–4,400 ng/ml (>10 years); ACTH, 9–50 pg/ml. Conversion to SI units: testosterone ng/ml×3.47 for nmol/l; cortisol µg/dl×27.6 for nmol/l; DHEAS ng/ml×2.56 for nmol/l; ACTH pg/ml×0.22 for pmol/l.DNA mutation numbering is based on the GenBank reference DNA sequence NM_004959.3 using current guidelines (www.hgvs.org/mutnomen) and with the A of the ATG initiation codon designated +1.

a Peak cortisol following synacthen stimulation.“At birth” refers to the first week of life. ACTH, adrenocorticotropin; NA, not analyzed.

### Case Histories

Patients 1–4 were diagnosed with ambiguous genitalia at birth. All these individuals presented with a similar genital phenotype consisting of phallic hypoplasia/clitoromegaly, fusion of the labioscrotal folds, a single perineal opening representing a urogenital sinus, and palpable inguinal gonads. The karyotype was found to be 46,XY in each case, but Müllerian structures (uterus, upper vagina) were absent. Gonadectomy–performed before the age of 2 years–revealed bilateral testes with Wolffian structures (epididymides, vasa deferentia). Endocrine evaluation at diagnosis showed low testosterone levels before and after human chorionic–gonadotropin (hCG) stimulation in Patients 1, 2 and 4, and low basal testosterone in Patient 3 (Table 1). In all patients, female gender assignment was made because of the significant undervirilization and clitoroplasty were undertaken following discussion and with parental consent.

Patient 5 presented as a young adult with primary amenorrhea. She had normal female external genitalia. Gonadotropins (folliclestimulating hormone [FSH] 111.9 U/l; luteinizing hormone [LH] 25.8 U/l) were elevated, karyotype was found to be 46,XY, and Müllerian structures and bilateral streak gonads were identified on ultrasound (Table 1).

Basal cortisol and dehydroepiandrosterone sulfate (DHEAS) were within normal ranges in Patients 1–4 and no symptoms or signs of adrenal insufficiency have emerged during follow–up (Table 1). A synacthen stimulation test performed at 17 years of age in Patient 2 showed a very good cortisol response.

### SF1 (NR5A1)Mutations

An overview of the SF1 (*NR5A1*) mutations detected is shown in Table 1 and Figure 1. Patients 1 and 2 harbor heterozygous missense mutations in SF1 (Patient 1: c.98G>C, p.C33S; Patient 2: _c_.251G>A, p.R84H), whereas Patient 3 has a heterozygous nonsense mutation (c.414C4A, p.Y138X). Patient 4 is heterozygous for a single nucleotide duplication (c.1277dupT), which is predicted to result in a frameshift and disruption of the ligand binding domain of SF1. Her mother also carries this heterozygous change. Patient 5 has a 4 base pair duplication (c.424_427dupCCCA) in exon 4 in one allele, which is predicted to result in a frameshift and premature stop codon after seven amino acids. A c.437G>C polymorphism (p.G146A) was present on the second allele, which was confirmed by subcloning the patient's DNA. Analysis of DNA from 100 healthy control subjects did not detect any of these key novel changes by restriction analysis (data not shown). The p.G146A change is a recognized nonsynonymous polymorphism in SF1 (rs1110061).

### Electromobility Shift Assays

**FIGURE 2:**
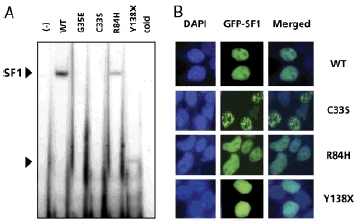
DNA binding, expression, and cellular localization of SF1. A: Electromobility shift assay showing wild–type (WT) and mutant SF1 binding to a labeled probe corresponding to the 3′ SF1 binding site of the *Cyp11a* promoter. in-vitro translated empty vector (−) (lane 1) and an excess of unlabeled cold probe (with WT SF1 protein) (lane 7) were used as controls. B:Cellular localization of GFP–SF1 fusion proteins (green), generated and expressed in tsa 201 cells using a pAcGFP–C1 vector. Nuclear counterstaining was performed with DAPI (blue), and images merged to conform nuclear localization.WT SF1 showed strong nuclear localization, with relative nucleolar exclusion and very occasional nuclear subfoci. A similar expression and localization pattern to WT was seen for the p.R84H mutant. Clustering in larger subnuclear foci was seen in the cells transfected with the p.C33S mutant.The p.Y138 Xmutant showed di¡use nuclear localization.

Studies of the 3′ *Cyp11a* promoter showed absent binding for the p.C33S mutant consistent with its position within the “P” box of the DBD. The p.R84H mutant and the p.Y138X truncated protein both showed reduced binding (Fig. 2A).

### SF1 Expression and Cellular Localization

GFP–tagged WT SF1 showed strong nuclear localization with nucleolar exclusion. A similar pattern was seen with the p.R84H mutant vector. The p.C33S construct showed marked subnuclear focal aggregation in many cells, whereas the p.Y138X nonsense mutant appeared to be located in a diffuse pattern throughout the nucleus (Fig. 2B).

### Functional Studies of SF1 Activity

**FIGURE 3:**
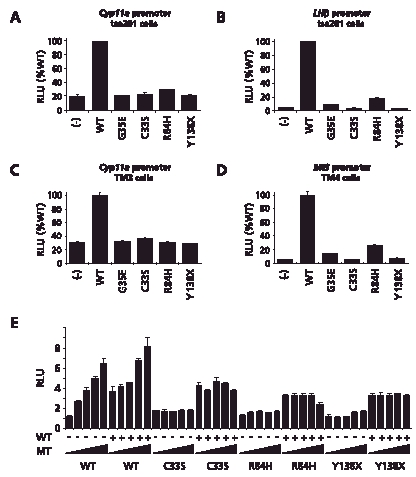
**A:** Effect of the SF1 mutants on transcriptional activity of the minimal promoters of *Cyp11a* in tsa 201 cells as described in themethods section. B: Effect of the SF1 mutants on synergistic activation of the *LH* beta promoter bySF1 and Egr1.C,D: Effect of the SF1 mutants on transcriptional activity of the *Cyp11a* and *MIS* promoter in TM3 (mouse Leydig) and TM4 (mouse Sertoli) cells, respectively. E: Studies of a potential dominant negative e¡ect ofmutant SF1 were performed by transfecting increasing amounts of wild–type (WT) ormutant SF1 (p.C33S, p.R84H, p.Y138X) expression vector (0,1,2,5,10 ng) with1–ng empty vector (−) or1–ngWT (1) and a *Cyp11a* promoter reporter (100 ng) in tsa 201cells. Additional empty vector was transfected to keep DNA quantities consistent.Data represent the mean 7± standard error of themean (SEM) of three independent experiments, each performed in triplicate.

All mutants studied (p.C33S, p.R84H, p.Y138X) showed markedly impaired transcriptional activity (Fig. 3A–D). Cotransfection of mutant with WT SF1 did not show a dominant negative effect even when 10:1 ratios of mutant:WT vector were transfected (Fig. 3E). The p.G146A polymorphism in SF1 has been shown previously to be associated with approximately 80% WT activity [WuQiang et al., 2003].

## DISCUSSION

Here, we present five novel heterozygous SF1 mutations in a cohort of 27 46,XY patients (18.5%) with severe underandrogenization but without adrenal insufficiency. These findings substantially increase the number of SF1 *(NR5A1)* mutations reported in humans, and show that mutations in SF1 can be found in patients with a range of phenotypic features (Table 2).

**TABLE 2:** Molecular and Clinical Features of 46, XY Individuals With in SF1*(NR5A1)*

Mutatiion	Occurence	Inheritance	Adrenal failure	External genitalia	Testicular dysgenesis	Müllerian structures	Reference
p.G35E	Heterozygous	De novo	+	Female	Severe	+	Achermann etal[1999]
p.R92Q	Homozygous	Recessive	+	Female	Not known	+	Achermann etal [2002]
c.424_427 dupCCCA+p.G146A	Heterozygous+ polymorphismxs	Not known	−	Female	Severe	+	This report
p.M781	Heterozygous	SLD	−	Female	Mild	(+)	Liin et al [2007]
p.V15M	Heterozygous	De novo	−	Female	Mild	−	Liin et al [2007]
p.C16X	Heterozygous	De novo	−	Ambiguous	Mild	+	Mallet et al [2004]
p.C91S	Heterozygous	SLD	−	Ambiguous	Mild	(+)	Lin et al [2007]
c.18delC	Heterozygous	De novo	−	Amdiguous	Mild	−	Has egawa et al [2004]
c.1058_1065del AGCTGGTG	Heterozygous	De novo	−	Ambiguous	Regressed^a^	−	Correa et al.[2004]
p.C33S	Heterozygous	De novo	−	Ambiguous	Mild	−	This report
p.R84H	Heterozygous	De novo	−	Ambiguous	Normal	−	This report
p.Y138X	Heterozygous	De novo	−	Ambiguous	Mild	−	This report
c.1277dupT	Heterozygous	SLD	−	Ambiguous	Normal	−	This report
p.L437Q	Heterozygous	De novo	−	Hypospadias	Mild	−	Lin et al.[2007]

a Gonadal tissue not found at laparoscopy at age 31 years

SLD, sex-limited dominant

+, present

−, absent

(+), remnant present

Within our series, four patients (heterozygous p.C33S, p.R84H, p.Y138X, and c.1277dupT) presented with a similar phenotype consisting of severe underandrogenization of the external genitalia, bilateral testes, and Wolffian structures, but absence of Muüllerian structures (uterus, fallopian tubes). These findings suggest that androgen biosynthesis and action were compromised during early fetal development (9–16 weeks), but that sufficient secretion of anti–Müllerian hormone (AMH) occurred from Sertoli cells to allow regression of Müllerian structures during this critical developmental period. The presence of well–formed Wolffian structures in Patients 1–4 is unusual given the degree of underandrogenization observed. Whether the development of Wolffian structures with relatively impaired androgenization of the external genitalia will be a feature that distinguishes SF1 mutations from other diagnoses, such as partial gonadal dysgenesis or partial androgen insensitivity, remains to be seen.

The identification of naturally–occurring missense mutations in SF1 is also helping to reveal important functional domains for nuclear receptor action. The amino acids of the missense mutations (p.C33S, p.R84H) are highly conserved in different species. The mutated cysteine (p.C33S) plays a central role in formation of the first zinc finger of the DNA binding domain of SF1 (Fig. 1). The C33S change results in impaired DNA binding to SF1 response elements (Fig. 2A), a complete loss of transcriptional activation without dominant negative activity (Fig. 3A–E) and abnormal clustering in sub7–nuclear aggregations (Fig. 2B). These foci likely represent promyelocytic leukemia (PML) or nuclear domain 10 (ND10) bodies, and have been reported previously with several nuclear receptor mutations associated with human disease [Baumann et al., 2001; Vottero et al., 2002; Black et al., 2004]. The arginine at position 84 lies between the zinc–fingers and “A”–box of SF1 in the carboxylterminal DNA–binding region (Fig. 1). This change also disrupts DNA binding and transcriptional activation, with little effect on cellular localization. Finally, the nonsense mutation (p.Y138X) results in a truncated protein with loss of the LBD, and the frameshift mutation resulting from the duplication c.1277dupT is predicted to lead to abnormal protein sequence. However, it is feasible that RNA containing the Y138X change is subject to nonsense–mediated decay.

Compared to the first four individuals, Patient 5 has a more severe phenotype, as she has normal female external genitalia and presented only in adolescence with absent breast development and primary amenorrhea. A uterus and streak gonads were found. The mutation c.424_427dupCCCA results in a frameshift and premature stop codon after seven amino acids. In addition to this heterozygous frameshift change, Patient 5 also harbors a heterozygous p.G146A polymorphism on the other allele. This p.G146A is reported to have approximately 80% of WT function and to be associated with micropenis or cryptorchidism [WuQiang et al., 2003; Wada et al., 2005, 2006]. Thus, we hypothesize that combination of the hypomorphic polymorphism, together with the heterozygous frameshift mutation, reduces gene dosage of SF1 below haploinsufficiency, resulting in the more severe phenotype observed in this patient.

While SF1 has a key role in testis development and function, the effects of reduced SF1 activity in the ovary are less clear [Hanley et al., 1999; Biason-Lauber and Schoenle, 2000]. The identification of a heterozygous frameshift mutation in SF1 in the mother of Patient 4 provides evidence that heterozygous SF1 mutations may be inherited in a sex–limited dominant fashion. Mothers may carry such changes, but have preserved ovarian function, whereas 46,XY offspring are affected. This finding has important implications for counseling, as heterozygous females may resemble “carriers” in an X–linked disorder.

Taken together, our findings show that heterozygous SF1 mutations are a relatively frequent finding in individuals with 46,XY DSD, and that the testis may be more sensitive to partial loss of SF1 function than the adrenal gland in humans [Jameson,2004; Lin et al., 2006]. Whether these patients will develop adrenal insufficiency with time remains to be seen. Thus, determining the genetic cause in 46,XY DSD can have implications for investigation, counseling of families, and longterm follow–up.
